# Baseline structural characteristics of the optic nerve head and retinal nerve fiber layer are associated with progressive visual field loss in patients with open-angle glaucoma

**DOI:** 10.1371/journal.pone.0236819

**Published:** 2020-08-20

**Authors:** Brent Siesky, Scott M. Wentz, Ingrida Januleviciene, Daniel H. Kim, Kendall M. Burgett, Alice C. Verticchio Vercellin, Lucas W. Rowe, George J. Eckert, Alon Harris

**Affiliations:** 1 Department of Ophthalmology, Icahn School of Medicine at Mount Sinai, New York, New York, United States of America; 2 Department of Ophthalmology, Indiana University School of Medicine, Indianapolis, Indiana, United States of America; 3 Eye Clinic of Medical Academy of Lithuanian University of Health Sciences, Kaunas, Lithuania; 4 University of Pavia, Pavia, Lombardy, Italy; 5 IRCCS—Fondazione Bietti, Rome, Lazio, Italy; 6 Department of Biostatistics, Indiana University School of Medicine, Indianapolis, Indiana, United States of America; Bascom Palmer Eye Institute, UNITED STATES

## Abstract

**Aims:**

To examine the relationship between baseline structural characteristics of the optic nerve head (ONH) and retinal nerve fiber layer (RNFL) and functional disease progression in patients with open-angle glaucoma (OAG) over 5 years.

**Methods:**

112 OAG patients were prospectively examined at baseline and every 6 months over a period of five years. Structural glaucomatous changes were examined with optical coherence tomography (OCT) and Heidelberg retinal tomography-III (HRT-III), and functional disease progression with automated perimetry (Humphrey visual fields). Cox proportional hazard models were used to assess the relationship between baseline structural measurements and functional disease progression.

**Results:**

From baseline over a 5-year period, statistically significant increases were found in OCT disc (D) area (p<0.001), cup (C) area (p<0.001), C/D area ratio (p<0.001), C/D horizontal ratio (p<0.001), C/D vertical ratio (p = 0.018), and a decrease in superior RNFL thickness (p = 0.008). Statistically significant increases were found in HRT-III C volume (p = 0.021), C/D area ratio (p = 0.046), mean C depth (p = 0.036), C shape (p = 0.008), and height variation contour (p = 0.020). Functional disease progression was detected in 37 of the 112 patients (26 of European descent and 11 of African descent; 33%). A statistically significant shorter time to functional progression was seen in patients with larger baseline OCT D area (p = 0.008), C area (p = 0.003), thicker temporal RNFL (p = 0.003), and in patients with a larger HRT-III C area (p = 0.004), C/D area ratio (p = 0.004), linear C/D ratio (p = 0.007), C shape (p = 0.032), or smaller rim area (p = 0.039), rim volume (p = 0.005), height variation contour (p = 0.041), mean RNFL thickness (p<0.001), or RNFL cross-sectional area (p = 0.002).

**Conclusion:**

Baseline ONH and RNFL structural characteristics were associated with a significantly shorter time to functional glaucomatous progression and visual field loss through the five-year period in OAG patients.

## Introduction

Primary open-angle glaucoma (OAG) is a multifactorial optic neuropathy characterized by progressive retinal ganglion cell death and a characteristic visual field (VF) loss [1]. Elevated intraocular pressure (IOP) has been identified as a major risk factor for OAG, and current treatments are limited to reducing and controlling IOP to arrest disease progression. Despite advances in pharmacological and surgical interventions, it is well established that glaucoma progression is still observed in some patients with IOP reduction. Additionally, a high percentage of individuals with ocular hypertension do not develop glaucoma [2–4]. These findings suggest that glaucomatous progression is multifactorial and other underlying factors contribute to disease onset and progression.

Many studies have been performed in order to identify IOP-independent risk factors for primary OAG. In particular, the presence of exfoliation, bilateral disease, advanced age, disc hemorrhages, thinner central corneas, lower systolic perfusion pressure, lower systolic blood pressure, and cardiovascular disease have been identified as predictors for glaucoma progression [5–7]. In addition, a history of migraine, female gender, increased vertical and horizontal cup-disc ratios, and pattern standard deviation were also found to be predictors for the development of glaucoma [5–8]. Many risk factors have been identified to help stratify at-risk patients within the population; however, specificity of biomarkers and their relative weight of contribution in the rate of progression is not currently well understood and severely limits physician management and intervention targets.

The multifactorial nature of glaucoma has led to the investigation of structural measurements as indicators of functional disease progression in glaucoma patients. Structural differences and changes to the ONH and RNFL have been shown to contribute to the onset and progression of OAG. Many studies, including the Ocular Hypertension Treatment Study, have found the vertical and horizontal cup-disc ratios to be strong structural predictors for the onset of OAG [8, 9]. Because of these findings, others have investigated the utility of various imaging modalities on determining structural differences between normal healthy eyes and those suffering from glaucoma [10–12]. These studies have found that baseline structural measurements, including RNFL thinning and cup shape, are associated with functional glaucomatous progression [13–16]. However, the results from these studies are varied, and the exact relationship between baseline structural characteristics of the ONH and RNFL and functional disease progression has not been fully delineated.

Many of these previous studies are limited in the specific structural measurements they investigate and vary in the statistical significance of their findings. Determining whether OAG patients will experience visual field changes affecting their quality of life is key in optimizing their treatment and care. Consequently, the development of predictive tools and/or models for glaucoma progression is a heavily-researched topic at this time [17, 18]. Currently, however, there is a paucity of information and tools that help glaucoma specialists in determining treatment. Here, we present data from a large, 5-year longitudinal study that aims to expand upon previous research and to evaluate baseline structural measurements that may predict which patients are at risk for functional glaucomatous progression after five years.

## Materials and methods

A cohort of 112 patients with OAG were enrolled at baseline, and prospectively examined at baseline and every 6 months over a period of five years at the Glaucoma and Diagnostic Center at Indiana University School of Medicine, Indianapolis, Indiana. Ethics approval was obtained by the Indiana University School of Medicine Institutional Review Board committee at the Indiana University School of Medicine. All patients signed an informed consent prior to initiation of this study, which adhered to the tenets of the Declaration of Helsinki. Our study was observational, and all medical management was performed by the patient’s physician and without regard to study participation. All participants were required to meet the following inclusion criteria: age 30 years or older and best-corrected visual acuity of 20/60 or better in the study eye. In addition, to be included, the clinical diagnosis of OAG had to be confirmed in the study eye by a fellowship-trained glaucoma specialist based upon criteria representative of glaucomatous optic disc or retinal nerve fiber layer (RNFL) structural abnormalities and/or automated perimetry visual field changes consistent with glaucomatous damage.

Patients were excluded for the following reasons: evidence of pseudoexfoliation or pigment dispersion, history of acute angle-closure glaucoma or a narrow occludable anterior chamber angle, history of chronic or recurrent inflammatory eye diseases, history of intraocular trauma, severe or progressive retinal disease, any abnormality preventing reliable applanation tonometry, cataract surgery within the past year, resting pulse < 50 beats per minute, or uncontrolled cardiovascular, renal, or pulmonary disease. Participants were allowed to continue their preventative blood pressure (BP) and cholesterol lowering medications. The data were categorized into groups of African Descent (AD) or European Descent (ED) based on self-reported race. Reporting of races other than AD or ED were excluded from this analysis.

One qualified eye was randomly designated as the observational study eye in each subject. All patients were questioned for their demographics, clinical history, ophthalmic history and medications, and systemic diseases and medications. Each subject was evaluated for heart rate (HR) and BP, which was assessed using an automated ambulatory blood pressure monitor after five minutes of rest. A comprehensive ophthalmological examination was performed including slit lamp evaluation, gonioscopy, central corneal thickness and axial length measurements, IOP measurement using Goldmann applanation tonometry, and indirect dilated ophthalmoscopy with a 90 diopters lens.

To limit reproducibility bias with imaging, a single experienced operator with over ten years of experience performed all measurements in the same order and at the same time of the day for each patient. Visual function was assessed by the Humphrey Field Analyzer II (HFA II), using the 24–2 Swedish Interactive Threshold Algorithm (SITA) standard (white III stimulus) version 4.1 (Carl Zeiss Meditec Inc, Dublin, CA). VF progression was determined using the Humphrey Glaucoma Progression Analysis (GPA) software [19]. Functional glaucoma progression was defined as 2 consecutive visits with an Advanced Glaucoma Index Study (AGIS) score increase ≥2 from baseline or a mean deviation (MD) decrease ≥2 from baseline.

RNFL thickness and optic nerve head (ONH) structures were assessed using both optical coherence tomography (OCT) (Stratus software V.4.0, Zeiss Meditec, Dublin, California, USA) and Heidelberg retinal tomography-III (HRT-III) (Heidelberg Engineering, Heidelberg, Germany) where HRT-III was utilized to supplement OCT evaluations for subtle RNFL and/or ONH changes. Measurements were made along a circle concentric with the optic disc (Fast RNFL Thickness acquisition protocol) to assess RNFL thickness. The RNFL thickness and cup/disc vertical and horizontal ratios were calculated using the device software [10–12, 18–21]. All participants baseline and follow up imaging was required to be of high quality, i.e. reliable visual fields with limited fixation losses and false-positive and false-negative results, and strong OCT and HRT III quality scores that were clinically valid to be included in our analysis. Statistical analysis involved performing a mixed-model analysis of covariance (ANCOVA) to test for significant change from baseline to five-year follow up. Factors associated with OAG functional progression were analyzed using Cox proportional hazards models in the PHREG program within SAS statistical software (SAS Institute Inc, Cary, NC); in detail, additional analyses explored the interaction of baseline OCT and HRT-III structural parameters with presence or absence of an increase in IOP of at least 3 mmHg from baseline to 5 years.

## Results

In this study, 112 patients with OAG were enrolled according to the prior listed inclusion and exclusion criteria. Overall baseline characteristics of the population revealed a mean age 64.9 ± 11.0 years; female (n = 68), male (n = 44); African descent (n = 29), European descent (n = 83); and non-insulin-dependent diabetes mellitus (n = 21), no diabetes mellitus (n = 91).

Table 1 shows the values for IOP, MD, AGIS score, OCT-measured average RNFL thickness, cup/disc horizontal ratio, and cup/disc vertical ratio at baseline and at five years. Each parameter had a statistically significant change when comparing measurements between the baseline and five-year values except for the average RNFL (p = 0.174). IOP significantly decreased at five years compared to baseline, while both the horizontal and vertical cup/disc ratios significantly increased. Functional visual field loss occurred in the patients, as there was a statistically significant decrease in the MD and a statistically significant increase in the AGIS score.

**Table 1:** Overall change in structural and functional measurements from baseline to five years.

	Baseline		5-yr		Change	
	N	Mean (95% CI)	N	Mean (95% CI)	Mean (95% CI)	P-value
IOP	111	16.68 (15.52, 17.84)	75	15.28 (14.01, 16.54)	-1.41 (-2.36, -0.45)	0.004*
MD	111	-3.38 (-4.43, -2.34)	78	-4.88 (-6.18, -3.57)	-1.49 (-2.35, -0.63)	0.001*
AGIS score	111	1.41 (0.86, 2.12)	77	2.14 (1.37, 3.16)	0.56 (0.28, 0.81)	< .001*
RNFL thickness average	111	74.91 (70.11, 79.70)	71	72.82 (67.59, 78.05)	-2.09 (-5.10, 0.92)	0.174
Cup/disc horizontal ratio	112	0.70 (0.65, 0.74)	73	0.76 (0.71, 0.80)	0.06 (0.03, 0.08)	< .001*
Cup/disc vertical ratio	112	0.684 (0.639, 0.730)	73	0.716 (0.666, 0.767)	0.032 (0.005, 0.059)	0.018*

AGIS: advanced glaucoma index score; CI: confidence interval; IOP: intraocular pressure; MD: mean deviation; N: number of patients who underwent the specific examination indicated in each row of the table at baseline and at 5 years; the number of patients who completed the study was not the same for different examinations, thus the difference between the “N” at baseline and after 5 years in the different rows of the table; RNFL: retinal nerve fiber layer.

*Bold P-value denotes a statistically significant difference between baseline and five-year follow up for all patients (p<0.05).

Table 2 displays the changes in ONH and RNFL structural measurements from baseline to five years obtained using OCT and HRT-III imaging.

**Table 2:** Overall change in OCT and HRT-III measurements from baseline to five years.

	Baseline		5-yr		Change	
	N	Mean (95% CI)	N	Mean (95% CI)	Mean (95% CI)	P-value
**OCT**						
Disc area	112	2.272 (2.141, 2.403)	73	2.618 (2.469, 2.766)	0.346 (0.249, 0.442)	< .001*
Cup area	112	1.184 (1.016, 1.352)	73	1.513 (1.324, 1.703)	0.329 (0.234, 0.425)	< .001*
Rim area	112	1.080 (0.950, 1.211)	73	1.104 (0.963, 1.246)	0.024 (-0.071, 0.119)	0.620
Cup/disc area ratio	112	0.514 (0.455, 0.574)	73	0.572 (0.509, 0.634)	0.058 (0.026, 0.090)	< .001*
Cup/disc horizontal ratio	112	0.70 (0.65, 0.74)	73	0.76 (0.71, 0.80)	0.06 (0.03, 0.08)	< .001*
Cup/disc vertical ratio	112	0.684 (0.639, 0.730)	73	0.716 (0.666, 0.767)	0.032 (0.005, 0.059)	0.018*
RNFL thickness superior	112	89.08 (82.24, 95.92)	71	82.85 (75.58, 90.13)	-6.23 (-10.80, -1.66)	0.008*
RNFL thickness inferior	112	91.32 (83.09, 99.55)	71	87.41 (78.10, 96.73)	-3.91 (-9.24, 1.42)	0.150
RNFL thickness nasal	112	63.15 (58.44, 67.86)	71	65.93 (60.15, 71.71)	2.78 (-1.58, 7.14)	0.211
RNFL thickness temporal	112	54.93 (50.02, 59.84)	71	54.44 (49.07, 59.81)	-0.49 (-3.90, 2.92)	0.779
RNFL average	112	74.91 (70.11, 79.70)	71	72.82 (67.59, 78.05)	-2.09 (-5.10, 0.92)	0.174
**HRT-III**						
Cup Area	111	0.870 (0.735, 1.015)	77	0.908 (0.768, 1.059)	0.037 (-0.001, 0.075)	0.057
Rim Area	111	1.268 (1.159, 1.377)	77	1.227 (1.115, 1.339)	-0.041 (-0.083, 0.002)	0.060
Cup Volume	111	0.296 (0.224, 0.368)	77	0.319 (0.246, 0.392)	0.023 (0.003, 0.042)	0.021*
Rim Volume	111	0.295 (0.246, 0.344)	77	0.292 (0.242, 0.341)	-0.003 (-0.022, 0.015)	0.719
Cup/Disc Area Ratio	111	0.410 (0.359, 0.461)	77	0.429 (0.377, 0.482)	0.019 (0.000, 0.038)	0.046*
Linear Cup/Disc Ratio	111	0.619 (0.573, 0.665)	77	0.632 (0.584, 0.679)	0.013 (-0.003, 0.029)	0.102
Mean Cup Depth	111	0.300 (0.266, 0.334)	77	0.309 (0.275, 0.343)	0.009 (0.001, 0.018)	0.036*
Max Cup Depth	111	0.724 (0.659, 0.790)	77	0.730 (0.665, 0.795)	0.006 (-0.015, 0.027)	0.581
Cup Shape	111	-0.128 (-0.149, -0.108)	77	-0.115 (-0.137, -0.092)	0.014 (0.004, 0.024)	0.008*
Height Variation Contour	111	0.330 (0.297, 0.366)	77	0.353 (0.316, 0.396)	0.022 (0.004, 0.040)	0.020*
Mean RNFL Thickness	111	0.196 (0.172, 0.219)	77	0.185 (0.159, 0.210)	-0.011 (-0.025, 0.003)	0.123
RNFL Cross-Sectional Area	111	1.025 (0.901, 1.148)	77	0.973 (0.839, 1.108)	-0.051 (-0.125, 0.022)	0.169

CI: confidence interval; HRT-III: Heidelberg retinal tomography-III; N: number of patients who underwent the specific examination indicated in each row of the table at baseline and at 5 years; the number of patients who completed the study was not the same for different examinations, thus the difference between the “N” at baseline and after 5 years in the different rows of the table; OCT: optical coherence tomography; RNFL: retinal nerve fiber layer.

*Bold P-value denotes a statistically significant difference between baseline and five-year follow up for all patients (p<0.05).

Table 3 condenses Table 2 into a solitary table where it highlights the structural parameters for each imaging modality that were shown to have a statistically significant change from the baseline to five-year measurements. Notably, not all of the structural parameters measured on OCT were measured on the HRT-III, and vice versa.

**Table 3:** Summary of statistically significant P-values for the change in structural parameters seen on OCT and HRT-III when comparing the baseline to five-year measurements for the study population.

Structural parameter	OCT	HRT-III
Disc area	< .001* **(↑)**	Not measured
Cup area	< .001* **(↑)**	0.057
Cud/disc area ratio	< .001* **(↑)**	0.0046* **(↑)**
Cup/disc horizontal ratio	< .001* **(↑)**	0.102
Cup/disc vertical ratio	0.018* **(↑)**	0.102
Mean cup depth	Not measured	0.036* **(↑)**
Cup shape	Not measured	0.008* **(↑)**
Superior RNFL thickness	0.008* **(↓)**	Not measured
Height variation contour	Not measured	0.020* **(↑)**
Mean RNFL thickness	0.174	0.123

OCT: optical coherence tomography; RNFL, retinal nerve fiber layer.

*Bold P-value denotes a statistically significant difference between baseline and five-year follow up for all patients (p<0.05). An arrow facing up (↑) means there was an increase in value when comparing baseline to five-year parameter values, whereas an arrow facing down (↓) means there was a decrease in value when comparing baseline to five-year parameter.

Functional disease progression was detected in 37 of the 112 patients (26 of European descent and 11 of African descent; 33%). In our study we did not find a statistically significant correlation between baseline central corneal thickness and functional progression after 5 years (p value = 0.8747).

Table 4 illustrates baseline ONH and RNFL structural measurements assessed by OCT and HRT-III and their relation to functional visual field loss over the five-year period.

**Table 4:** Baseline ONH and RNFL structural measurements by OCT and HRT-III and their relation to functional visual field loss over the five-year period.

	Functional Progression after 5 years	No Functional Progression after 5 years	Hazard Ratio (95% CI)	P-value
	[N = 37] Mean (SE)	[N = 75] Mean (SE)		
**OCT**				
Disc area	2.403 (0.092)	2.180 (0.046)	1.53 (1.12–2.09) for difference of 0.5	0.008*
Cup area	1.398 (0.110)	1.110 (0.059)	1.48 (1.14–1.92) for difference of 0.5	0.003*
Rim area	1.004 (0.072)	1.070 (0.054)	0.83 (0.58–1.19) for difference of 0.5	0.311
Cup/disc area ratio	0.566 (0.034)	0.512 (0.025)	1.28 (0.95–1.74) for difference of 0.2	0.108
Cup/disc horizontal ratio	0.75 (0.03)	0.70 (0.02)	1.45 (0.95–2.23) for difference of 0.2	0.084
Cup/disc vertical ratio	0.726 (0.026)	0.685 (0.019)	1.42 (0.92–2.19) for difference of 0.2	0.113
RNFL thickness superior	84.05 (4.05)	89.77 (2.92)	0.80 (0.57–1.11) for difference of 25	0.183
RNFL thickness inferior	84.97 (4.07)	92.92 (3.53)	0.78 (0.58–1.05) for difference of 25	0.108
RNFL thickness nasal	63.78 (3.77)	60.19 (2.11)	1.17 (0.78–1.75) for difference of 25	0.445
RNFL thickness temporal	53.24 (2.31)	61.43 (1.91)	0.61 (0.44–0.84) for difference of 15	0.003*
Mean RNFL thickness	71.52 (2.61)	76.07 (2.08)	0.78 (0.59–1.05) for difference of 15	0.097
**HRT-III**				
Cup Area	1.094 (0.093)	0.830 (0.051)	2.50 (1.34–4.68) for difference of 0.5	0.004*
Rim Area	1.151 (0.059)	1.298 (0.049)	0.62 (0.40–0.98) for difference of 0.5	0.039*
Cup Volume	0.331 (0.050)	0.243 (0.026)	1.32 (0.97–1.79) for difference of 0.3	0.077
Rim Volume	0.222 (0.019)	0.314 (0.021)	0.46 (0.29–0.79) for difference of 0.2	0.005*
Cup/Disc Area Ratio	0.469 (0.029)	0.382 (0.020)	1.79 (1.20–2.65) for difference of 0.2	0.004*
Linear Cup/Disc Ratio	0.672 (0.023)	0.595 (0.019)	2.01 (1.21–3.34) for difference of 0.2	0.007*
Mean Cup Depth	0.293 (0.020)	0.284 (0.015)	1.11 (0.68–1.81) for difference of 0.2	0.678
Max Cup Depth	0.684 (0.034)	0.701 (0.030)	0.93 (0.63–1.36) for difference of 0.3	0.699
Cup Shape	-0.112 (0.013)	-0.134 (0.008)	1.70 (1.05–2.75) for difference of 0.1	0.032*
Height Variation Contour	0.317 (0.017)	0.376 (0.016)	0.84 (0.71–0.99) for difference of 0.2	0.041*
Mean RNFL Thickness	0.156 (0.011)	0.208 (0.009)	0.43 (0.27–0.69) for difference of 0.1	<0.001*
RNFL Cross-Sectional Area	0.825 (0.057)	1.074 (0.051)	0.50 (0.32–0.77) for difference of 0.5	0.002*

HRT-III: Heidelberg retinal tomography-III; N: number of patients; OCT: optical coherence tomography; ONH: optic nerve head; RNFL: retinal nerve fiber layer; SE: Standard Error.

*Bold P-value denotes a statistically significant difference between patients who progressed and those who did not progress (p<0.05).

Cox proportional hazards models show that baseline OCT measurements in patients with a larger disc area (Hazard ratio, HR 1.53 for a disc area difference of 0.5; p = 0.008), a larger cup area (HR 1.48 for 0.5 difference; p = 0.003), or a thinner RNFL temporal thickness (HR 1.63 for 0.5 difference; p = 0.003) were associated with a statistically significant shorter time to functional disease progression.

Similarly, certain baseline HRT-III measurements in these patients were also associated with a shorter time to functional disease progression. These baseline HRT-III measurements include a larger cup area (HR 2.50 for a cup area difference of 0.5; p = 0.004), a greater cup/disc area ratio (HR 1.79 for 0.2 difference; p = 0.004), a greater linear cup/disc ratio (HR 2.01 for 0.2 difference; p = 0.007), a greater cup shape value (HR 1.70 for 0.1 difference; p = 0.032), a smaller rim area (HR 1.60 for 0.5 difference; p = 0.039), a smaller rim volume (HR 2.17 for 0.2 difference; p = 0.005), a smaller height variation contour (HR 1.19 for 0.2 difference; p = 0.041), a smaller RNFL cross-sectional area (HR 2.02 for 0.5 difference; p<0.001), or a thinner mean RNFL thickness (HR 2.32 for 0.1 difference; p = 0.002).

Table 5 is a condensed version of Table 4 showing only the parameters that were statistically significant for each imaging modality. Notably, not all of the structural parameters measured on OCT were measured on the HRT-III and vice versa.

**Table 5:** Summary of statistically significant HR and P-values from differences in baseline values for various structural parameters acquired by OCT and HRT-III between patients who progressed at 5 years and those who did not.

Structural parameter	OCT	HRT-III
Disc area	HR 1.53; p = 0.008* **(↑)**	Not measured
Cup area	HR 1.48; p = 0.003* **(↑)**	HR 2.50; p = 0.004* **(↑)**
Cup/disc area ratio	0.108	HR 1.79; p = 0.004* **(↑)**
Linear cup/disc ratio	Not measured	HR 2.01; p = 0.007* **(↑)**
Cup shape	Not measured	HR 1.70; p = 0.032* **(↑)**
Rim area	0.311	HR 1.60; p = 0.039* **(↓)**
Rim volume	Not measured	HR 2.17; p = 0.005* **(↓)**
Height variation contour	Not measured	HR 1.19; p = 0.041* **(↓)**
Mean RNFL thickness	0.097	HR 2.32; p< 0.001* **(↓)**
Temporal RNFL thickness	HR 1.63; p = 0.003* **(↑)**	Not measured
RNFL cross-sectional area	Not measured	HR 2.02; p = 0.002* **(↓)**

HR: hazard ratio; HRT-III: Heidelberg retinal tomography-III; OCT: optical coherence tomography; RNFL: retinal nerve fiber layer.

*Bold P-value denotes a statistically significant difference between baseline and five-year follow up for all patients (p<0.05). An arrow facing up (↑) means a larger baseline value was associated with functional progression, whereas an arrow facing down (↓) means a lower baseline value was associated with functional progression at 5 years; insignificant HRs were omitted.

Additional analyses explored the interaction of baseline OCT and HRT-III structural parameters with presence (12) or absence (63) of an increase in IOP of at least 3 mmHg over the five years on time to progression. Baseline OCT measurements for cup area (p = 0.048) and cup/disc area ratio (p = 0.022) were more strongly associated with shorter time to functional disease progression in patients with increased IOP than in patients who did not experience an increase in IOP. Many other OCT and HRT-III parameters displayed similar differences between the two groups, but the differences did not reach statistical significance.

## Discussion

In this study, we tracked ONH and RNFL structure over a five-year period using OCT and HRT-III imaging in order to determine if baseline structural parameters may be associated with a shorter time to functional progression in patients who already had a diagnosis of open-angle glaucoma. As progressive OAG represents an increasing impact on quality of life through an ageing population, advancing specificity in diagnostic outcomes will significantly affect patient quality of life [22]. Our results strongly support previous investigations that have identified baseline ONH and RNFL characteristics that are associated with shorter time to functional glaucoma progression. Furthermore, our results identify additional structural characteristics that are associated with a shorter time to functional glaucoma progression.

Specifically, our findings showed that patients with a larger baseline disc area, a larger cup area, a larger cup/disc area ratio, or a larger linear cup/disc ratio were associated with shorter time to functional glaucoma progression. These findings are supported by previous reports from the Ocular Hypertension Treatment Study that found that the ONH vertical and horizontal cup-disc ratios were structural parameters strongly associated with the onset of OAG [8]. In addition to cup/disc ratios, the evaluation of the RNFL thickness by OCT and HRT-III can be useful in the clinical setting, as certain RNFL characteristics were associated with a shorter time to functional disease progression over the course of five years. Similar to our findings, Sehi *et al*. showed that progressive RNFL loss was associated with functional glaucoma progression [13], while Lalezary *et al*. showed that thinner baseline RNFL, as measured by OCT, was an independent predictor of functional glaucomatous changes [14]. Our data also show that baseline optic disc cup shape, based on measures of the topography including cup size, cup depth, neuroretinal rim slope, and RNFL curvature, appears to be a significant predictor of glaucoma progression. In a study of 80 patients with exfoliation syndrome, Harju and Vesti reported that among HRT parameters, only cup shape measurements showed statistically significant change in MD [15]. Similarly, in a study of 68 eyes with OAG, Saarela and Airaksinen found that cup shape was the only optic nerve head parameter that showed a statistically significant correlation with progression of an RNFL defect [16]. Interestingly, our data shows an increase in disc area and rim area when measured by OCT. In the Baltimore study, similar results regarding increase in disc area were found although they were unable to find statistical significance [23]. Unlike previous studies such as the one performed by Hammel *et al*., our OCT measurements found an increase in rim area, which is unusual [24]. Taken together, these research findings may be useful for the development of prediction models which could utilize baseline structural change in newly-diagnosed OAG patients to determine whether patients are likely to progress, the rate at which they may progress, and if they require earlier intervention (i.e., pharmacologic, procedural, and/or operative) at the time of diagnosis. Such models would greatly simplify the decision-making process for clinicians who manage patients with OAG.

We also explored as to whether patients who experienced an increase in IOP over the five years displayed different associations between the baseline structural parameters and functional disease progression. The patients were categorized as having increased IOP if the IOP increased by at least 3 mmHg from baseline to 5 years. This was determined to be the smallest value to use for defining an IOP increase given the variability in the IOP measurements. It is worth noting, however, that only 12 of the 75 patients with 5-year changes in IOP had an increase of 3 mmHg or more. These results indicate a possible difference in the risk for functional disease progression associated with baseline structure with versus without subsequent changes in IOP, but a larger study would be needed to further clarify this difference.

This study has several strengths, such as having a relatively large study population prospectively evaluated over a long period of time, a single imaging data collector to limit reproducibility bias, and stringent exclusion criteria. However, we recognize that our study is not without limitations. Our study lacks a control group of healthy, non-glaucoma patients; thus, our ability is limited in drawing correlations between structural and functional changes that may be seen in healthy controls. Since there were no criteria established for excluding patients with advanced disease at baseline, there is a chance of introducing bias. Additionally, participation in this study did not affect glaucoma treatment; thus, patients were on different types and amounts of ocular antihypertensives throughout the course of this study. Additionally, many patients were also on systemic antihypertensives, which could have an effect on disease progression due to differences in ocular perfusion pressure status [25].

In conclusion, this study revealed specific prognostic baseline structural characteristics of the ONH and RNFL that were associated with functional glaucoma progression. These data may help in determining algorithms with higher specificity to predict glaucoma risk and risk for progression in patients with glaucoma.

## Supporting information

S1 Fig Survival function for time to progression for baseline disc area and cup area measured with optical coherence tomography. Lines represent survival curves for lowest and highest observed values for each measurement. Higher baseline disc area and cup area were associated with shorter time to functional progression. HR-hazard ratio.
(PDF)

S2 Fig Survival function for time to functional progression for baseline linear cup/disc ratio, cup area, cup shape, rim area, rim volume, height variation contour, mean retinal nerve fiber layer (RNFL) thickness, and RNFL cross-sectional area measured with Heidelberg retinal tomography-III. Lines represent survival curves for lowest and highest observed values for each measurement. Higher baseline linear cup/disc ratio, cup area, and cup shape and lower baseline rim area, rim volume, height variation contour, mean RNFL thickness, and RNFL cross-sectional area were associated with shorter time to functional progression.
(PDF)
