# Supplementary figures and images for: Baseline structural characteristics of the optic nerve head and retinal nerve fiber layer are associated with progressive visual field loss in patients with open-angle glaucoma
Source: PLoS One. 2020 Aug 20;15(8):e0236819. doi: 10.1371/journal.pone.0236819 (PMC7444539; doi:10.1371/journal.pone.0236819)

### Disc area

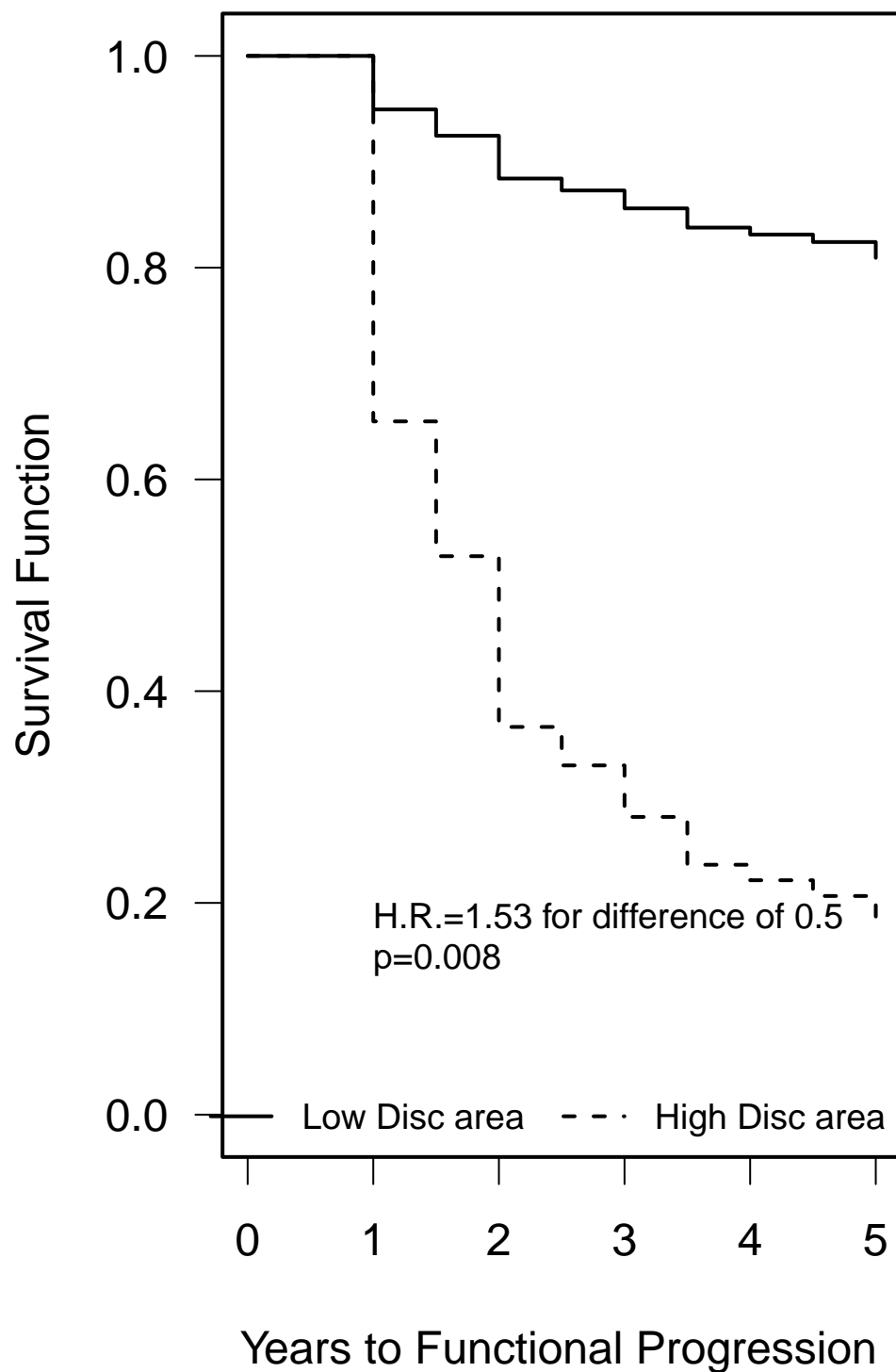

### Cup area

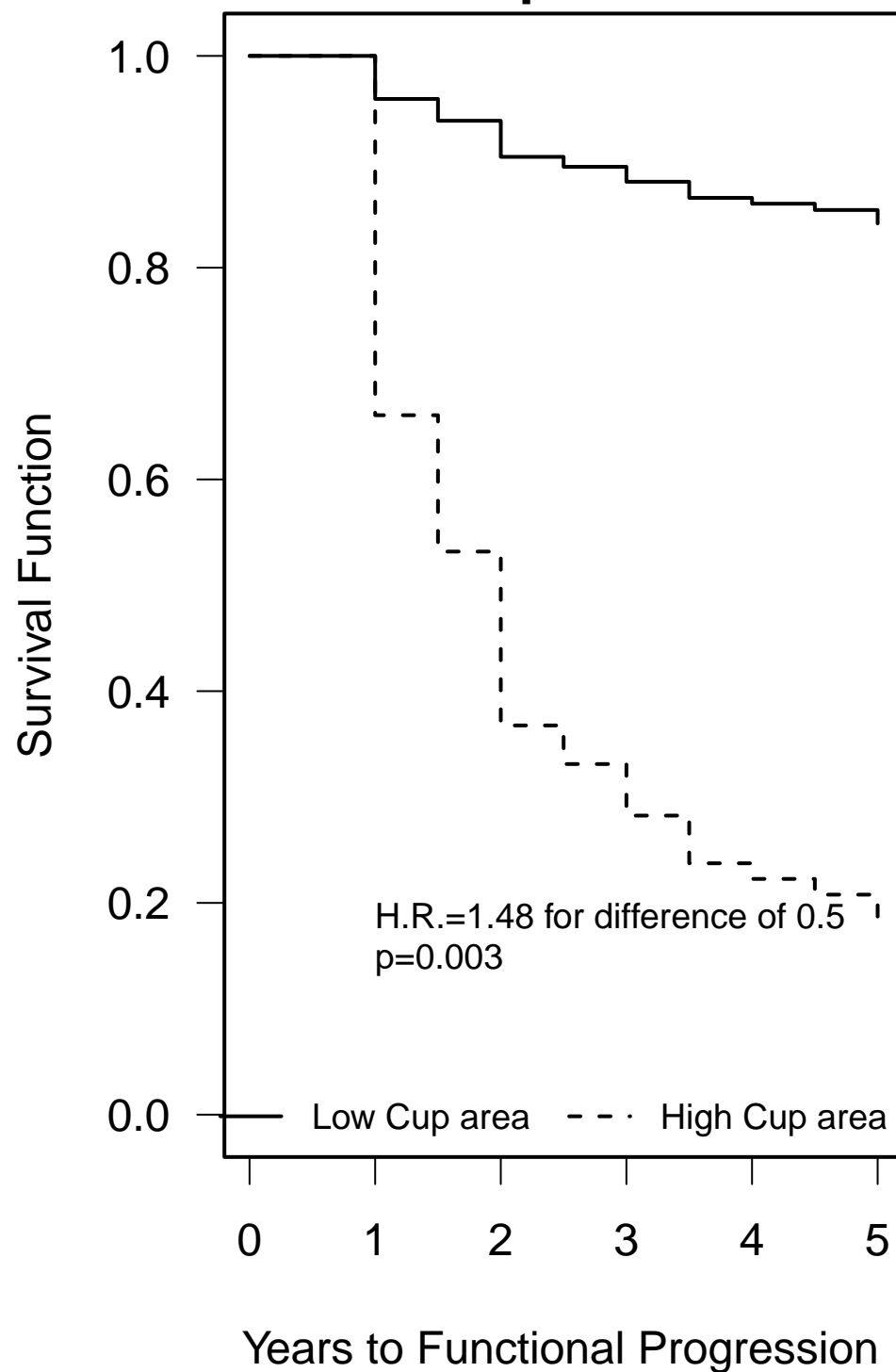

Supplement: S1 Fig — Lines represent survival curves for lowest and highest observed values for each measurement. Higher baseline disc area and cup area were associated with shorter time to functional progression. HR-hazard ratio. (PDF) [file pone.0236819.s001.pdf]

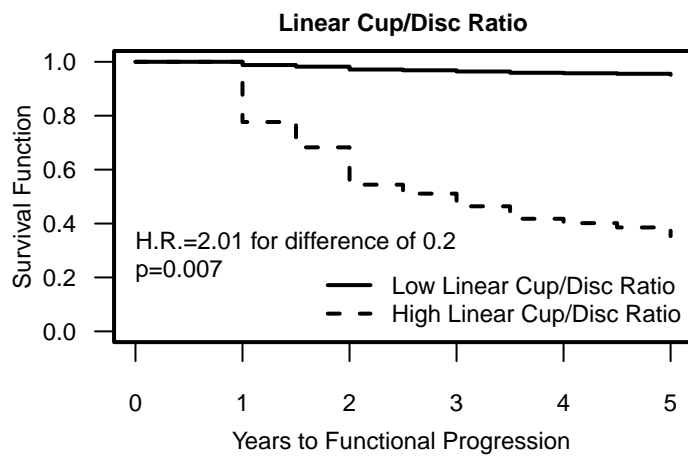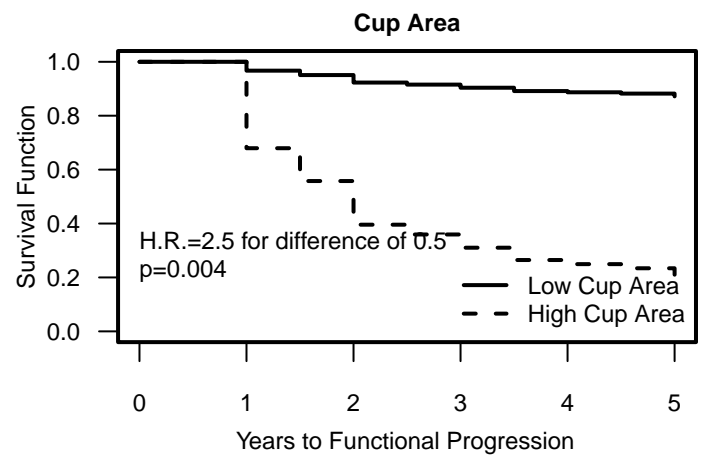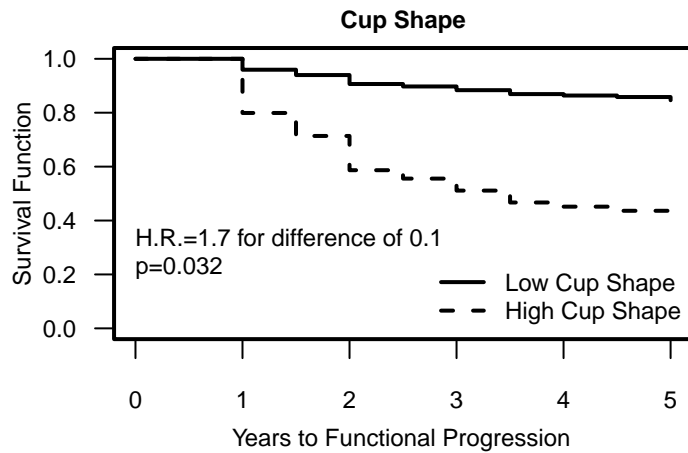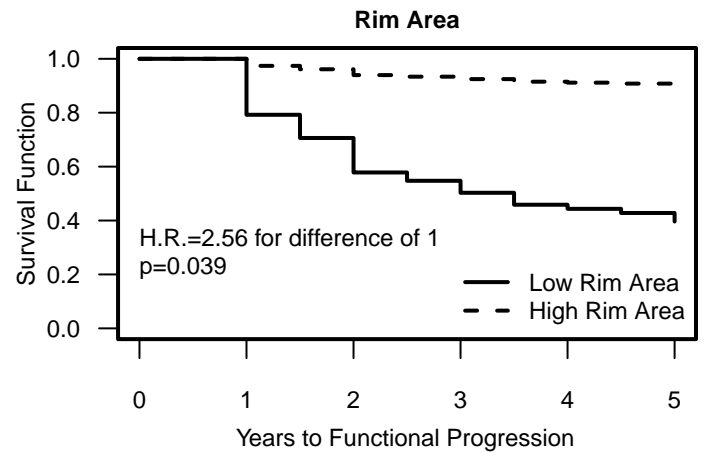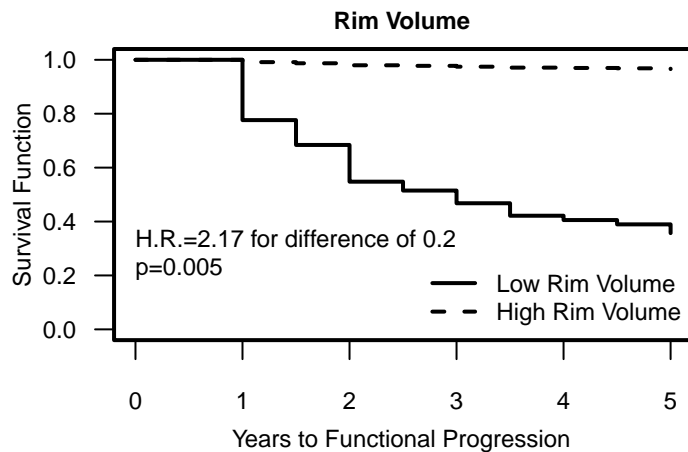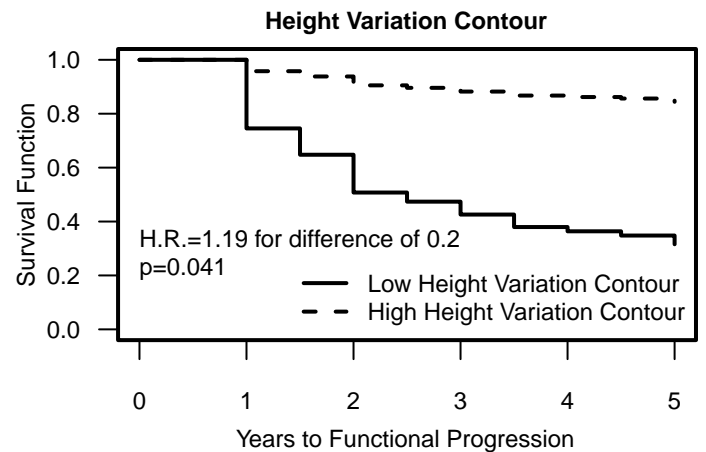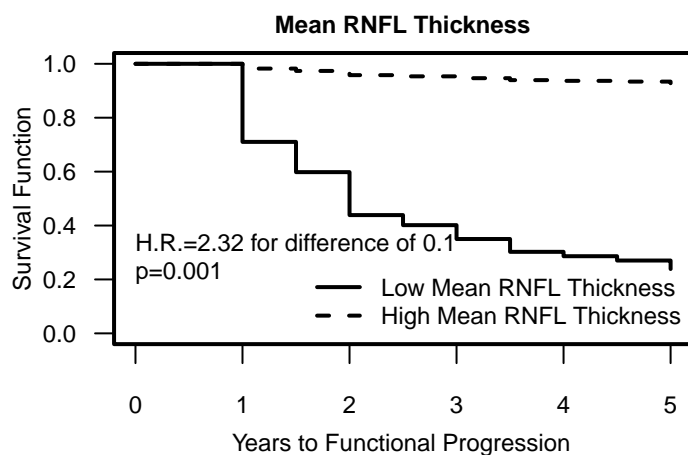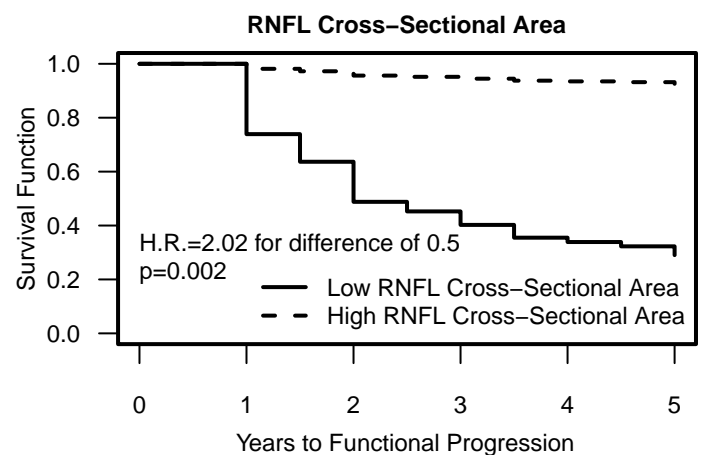

Supplement: S2 Fig — Lines represent survival curves for lowest and highest observed values for each measurement. Higher baseline linear cup/disc ratio, cup area, and cup shape and lower baseline rim area, rim volume, height variation contour, mean RNFL thickness, and RNFL cross-sectional area were associated with shorter time to functional progression. (PDF) [file pone.0236819.s002.pdf]
